# The Role of Triboloading Conditions in Tribolayer Formation and Wear Resistance of PES-Based Composites Reinforced with Carbon Fibers

**DOI:** 10.3390/polym16152180

**Published:** 2024-07-31

**Authors:** Defang Tian, Changjun He, Dmitry G. Buslovich, Lyudmila A. Kornienko, Sergey V. Panin

**Affiliations:** 1 Department of Materials Science, Engineering School of Advanced Manufacturing Technologies, National Research Tomsk Polytechnic University, 634050 Tomsk, Russia; defan1@tpu.ru (D.T.); chanczyun1@tpu.ru (C.H.); 2 Laboratory of Nanobioengineering, Institute of Strength Physics and Materials Science of Siberian Branch of Russian Academy of Sciences, 634055 Tomsk, Russia; buslovich@ispms.ru; 3 Laboratory of Mechanics of Polymer Composite Materials, Institute of Strength Physics and Materials Science of Siberian Branch of Russian Academy of Sciences, 634055 Tomsk, Russia; rosmc@ispms.ru

**Keywords:** polyethersulfone (PES), wear, short carbon fibers (SCFs), tribological layer, friction coefficient, dry sliding friction

## Abstract

In this paper, the tribological characteristics of polyethersulfone-based composites reinforced with short carbon fibers (SCFs) at aspect ratios of 14–250 and contents of 10–30 wt.% are reported for linear metal–polymer and ceramic–polymer tribological contacts. The results showed that the wear resistance could be greatly improved through tribological layer formation. Loading PES with 30 wt.% SCFs (2 mm) provided a minimum WR value of 0.77 × 10^−6^ mm^3^/N m. The tribological layer thicknesses were estimated to be equal to 2–7 µm. Several conditions were proposed, which contributed to the formation of a tribological layer from debris, including the three-stage pattern of the changing kinetics of the time dependence of the friction coefficient. The kinetics had to sharply increase up to ~0.4–0.5 in the first (running-in) stage and gradually decrease down to ~0.1–0.2 in the second stage. Then, if these levels did not change, it could be argued that any tribological layer had formed, become fixed and fulfilled its functional role. The PES-based composites loaded with SCFs 2 mm long were characterized by possessing the minimum CoF levels, for which their three-stage changing pattern corresponded to one of the conditions for tribological layer formation. This work provides valuable insight for studying the process parameters of tribological layer formation for SCF-reinforced thermoplastic PES composites and revealing their impact on tribological properties.

## 1. Introduction

Polyethersulfone (PES), a member of the polysulfone class, is a high-performance polymer (HPP) with a high glass transition temperature (Tg) of 225 °C and an operating, temperature of up to 180 °C. PES exhibits relatively high strength and elasticity modulus, long fatigue life and high dimensional stability. It is also resistant to harsh environments, fire, radiation, etc. [1]. While PES-based composites reinforced with various fibers exhibit excellent processability and enhanced mechanical properties [2], their application in industrial (metal–polymer) friction units has been limited. This fact is primarily attributed to the low wear resistance of PES, which is linked to its specific chemical structure [3].

Various methods can be employed to improve the wear resistance of PES, including loading of nano- and micro-scale fillers, chemical modification, surface treatment with ionizing radiation, mechanical activation and others [4]. The challenge of enhancing tribological characteristics can be addressed by incorporating solid lubricant fillers, which reduce both wear rate (WR) values and the coefficient of friction (CoF) levels. Among the most common and cost-effective solid lubricant materials for polymer matrix composites (PMCs) are polytetrafluoroethylene (PTFE) [5,6], molybdenum disulfide (MoS_2_) [7], etc. Carbon is frequently employed as a filler in PMCs due to the abundance of its forms, including carbon fibers (CFs), graphite (Gr), carbon black (CB), graphene nanoparticles (nGrs) and carbon nanotubes (CNTs) [8,9,10,11]. These forms are widely utilized in the design of polymer matrix composites for diverse applications. Traditionally, these micro-fillers are employed to enhance the thermal, electrical and mechanical properties of neat polymers [12,13]. In particular, short CFs (SCFs) are widely employed in the reinforcement of polymer composites due to their high specific strength, elastic modulus, electrical conductivity, tribological characteristics and resistance to heat and acids [14].

A review article [15] highlights that, in polymer composites, besides other factors, the tribological characteristics depend on the chemical structure of the components and the type of tribological contact (point, line or plane). Therefore, when developing PES-based composites for various operating conditions, including friction units, it is crucial to simultaneously achieve optimal tribological characteristics while maintaining high mechanical properties through the selection of optimal fillers. 

As reported in [16,17], the wear resistance of high-strength HPP-based composites can be improved (up to WR values of ~10^−6^ mm^3^/N·m) by implementing an adhesion wear mechanism in both linear and planar tribocontacts under dry sliding friction conditions against steel counterfaces (in pin-on-disk or block-on-ring schemes) with an *Ra* surface roughness of 0.1–0.3 μm. To achieve this, it is necessary to (i) suppress (minimize) the chemical interaction between the rubbing parts (which can be accomplished, among other things, by forming and adhering a transfer film), (ii) maintain a low surface roughness (of the run-in) of the sliding surface of the polymer composite for a long time, including loading with particles of the solid lubricants (which additionally ensure a low coefficient of friction) and (iii) form a tribological layer on a wear track surface. The effect of this process is similar to a callus on a human/animal body, providing protection against the plowing action of asperities on the counterpart surface.

Currently, SCFs are one of the most common and effective types of fillers for HPP-based composites. They can act as a single filler with a content ranging from 10 to 30 wt.%, or be part of complex fillers, along with, for example, PTFE and/or graphite (so-called high-pressure-velocity (HPV) composites). There is a considerable number of papers dedicated to studying the influence of carbon fiber loading on the tribological properties of composites based on PEEK [18,19,20], PPS [21,22,23,24] and PI [25,26,27,28]; however, studies focusing on PES are relatively scarce. 

Zhao Z.K. et al. investigated the effect of incorporating 5–30% SCFs and short glass fibers (SGFs) on the tribological characteristics of PES composites [29]. Their findings demonstrated that loading PES with ≥20% SCFs reduced CoF levels and WR values by 1.5 and 14 times, respectively. This effect was attributed to the fact that when SGF/PES and SCF/PES composites experience wear, SGFs and SCFs bear the shearing load during sliding, significantly mitigating the wear of the PES composites.

Bijwe J. et al. [30] fabricated PES-based composites reinforced with SCF at 30% and 40 wt.% SCFs. Their research showed that incorporating 40 wt% of carbon fibers yielded optimal adhesive wear characteristics (WR~10-16 m^3^/Nm compared to WR~10-13 m^3^/Nm for pure PES). During the wear process of this reinforced composite, carbon fibers primarily bear the load during sliding. This effectively inhibits the wear of the polymer matrix, as carbon fibers possess superior strength and wear resistance, significantly limiting the overall wear of the polymer matrix.

Zhao W.Y. et al. varied the contents of SCFs from 5 to 35% and their lengths from 40 to 100 µm [31]. Loading PES with SCFs improved the wear resistance of the composites. SCFs 100 µm long were superior to those with a length of 40 µm, lowering both CoF levels and WR values significantly. At the SCF_100µm_ content of 15%, they were 0.15 and 1 × 10^−5^ mm^3^/N·m, respectively, in contrast to 0.20 and 3 × 10^−5^ mm^3^/N·m for the shorter ones.

This study aims to enhance the tribological properties (specifically wear resistance) of PES-based composites under linear tribological contacts in a metal–polymer interface through reinforcement with SCFs with an aspect ratio (AR) of 14–250. This study will vary the content of SCF and investigate the role of tribological loading conditions in the formation of the tribolayer and evolution for polymeric composites with a PES matrix that is not chemically inert, a key factor determining wear resistance. 

## 2. Materials and Methods

### 2.1. Materials

For the fabrication of composites, PES “Veradel^®^ 3100” powder with a particle size of 10 µm was purchased from “Solvay” (“Solvay”, Brussels, Belgium). The powder was loaded with the commercially available fillers, as presented in Table 1. 

The composites were loaded with commercially available SCFs, presented in Table 2, at contents of 10, 20 and 30 wt.%.

### 2.2. Fabrication of the Composites

The PES powder and the fillers were mixed by dispersing the suspension components in alcohol using a “PSB-Gals 1335-05” ultrasonic cleaner (“PSB-Gals” Ultrasonic equipment center, Moscow, Russia). The processing duration was 3 min at a generator frequency of 22 kHz. After mixing, the suspensions were dried in a “Memmert UF110” oven (Memmert GmbH + Co. KG, Schwabach, Germany) with forced ventilation for 3 h at a temperature of 120 °C. The PES-based composites were fabricated by hot pressing at a pressure of 15 MPa and a temperature of 370 °C with a subsequent cooling rate of 2 °C/min. The samples were cut from the plates using a CNC milling machine. The required surface quality of the specimens was achieved by grinding with sandpapers up to P2000 (ISO 6344) [32].

### 2.3. Physical and Mechanical Properties

Tensile properties of dog-bone-shaped type V samples (according to ASTM D638) [33] were assessed using an “Instron 5582” electromechanical testing machine (Instron, Norwood, MA, USA). The number of samples of each composite type was at least four. The total length of the specimens was 64 mm, the gauge length was 10 mm and the cross-sectional area was 3.2 mm × 3.2 mm. The strains were measured by a non-contact (optical) method with a “VIC 3D” setup, implementing the Digital Image Correlation (DIC) method (Correlated Solutions, Irmo, SC, USA). Static tension tests were conducted at a cross-head speed of 1 mm/min, according to ASTM D638 [33].

### 2.4. Tribological Characteristics

In the linear tribological contact, according to the “block-on-ring” scheme (ASTM G77-17) [34], dry sliding friction tests were performed using a “2070 SMT-1” friction testing machine (Tochpribor Production Association, Ivanovo, Russia). The *P* load was 60 N, while the *V* sliding speed was 0.3 m/s.

Metal counterfaces were made of the outer ring of a commercial bear (the GCr15 grade) as well as from AISI 321 austenitic stainless steel, while a ceramic one was manufactured from Al_2_O_3_. They had a disk shape with an outer diameter of 35 mm and a width of 11 mm. The *Ra* surface roughness of the counterfaces was 0.20–0.25 µm and was evaluated with a ‘New View 6200’ profilometer (Zygo, Middlefield, CT, USA).

During the tribological tests, the counterface temperatures were assessed with a “CEM DT-820” non-contact infrared thermometer (Shenzhen Everbest Machinery Industry Co., Ltd., Shenzhen, China).

WR values were determined by measuring widths and depths of wear tracks by stylus profilometry (KLA-Tencor, Milpitas, CA, USA), followed by multiplication by their length. They were calculated taking into account both *P* load and *l* distance values:

(1) $$Wear\;rate=\frac{volume\;loss\;(mm^{3})}{load\left(N\right)\times sliding\;distance\;(m)}$$

### 2.5. Structural Studies

The topography of the wear track surfaces was studied using a “Neophot 2” optical microscope (Carl Zeiss, Jena, Germany) equipped with a “Canon EOS 550D” digital camera (Canon Inc., Tokyo, Japan) and an “Alpha-Step IQ” stylus profiler (KLA-Tencor, Milpitas, CA, USA). The latter made it possible to measure the *Ra* surface roughness of the wear track surfaces as well.

Chemical compositions of the tribological layers were analyzed using Raman spectroscopy with a “Renishaw inVia Basis Raman” spectrometer (Renishaw plc, Gloucestershire, UK).

Before scanning electron microscopy (SEM) examinations, carbon films were deposited on the specimens with a “Quorum Technologies EMITECH K450X” setup (Quorum Technologies, Laughton, UK). To investigate the structures in the subsurface layers after the end of tribological tests, an “Apreo 2 S” scanning electron microscope (Thermo Fisher Scientific, Waltham, MA, USA) was used at accelerated voltages of 10, 20 and 30 kV and an e-beam current of 0.80 nA.

## 3. Results

### 3.1. Mechanical Properties

**Figure 1:**
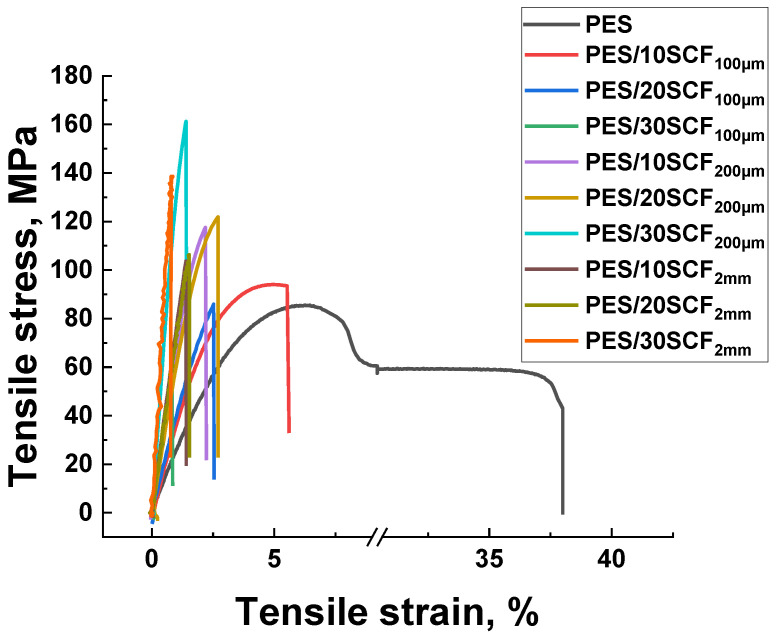
The stress–strain diagrams of neat PES and PES/SCF composites.

Table 3 and Figure 1 present the physical and mechanical properties of neat PES and its composites with different contents of SCFs. According to these data, the modulus of elasticity E increased by 2–6 times when the polymer was filled with 10–30 wt.% SCFs (indicating their effectiveness as a strengthening filler), while the ultimate tensile strength was enhanced by 10–17% and the elongation at break decreased by 9–40 times. As expected, enhancing the content of SCFs was accompanied by embrittlement of the composites (for example, elongation at break did not exceed 0.86% at 30 wt.%).

### 3.2. The Linear Tribological Contact

Table 4 presents the tribological characteristics of neat PES and the PES-based composites determined in the linear tribological contact (according to the ‘block-on-ring’ scheme) against the GCr15 steel counterface. For all studied composites, no significant increase in temperature at the tribocontact was observed under the metal–polymer rubbing conditions compared to neat PES. This fact reflected, in part, the high thermal conductivity of the GCr15 steel counterface, which effectively dissipated the frictional heat generated by overcoming the friction force. Loading PES with SCFs 100 µm long at AR of ~10 did not reduce WR values, while CoF levels only slightly decreased to a level of ~0.4–0.5.

According to the authors of [35], lowering CoF levels to ~0.2 could indicate the tribological layer formation on HPP-based composites, which are characterized by high strength. This should reduce the CoF values under linear tribological contacts. This phenomenon contributed to a significant reduction in WR compared to neat PES. The authors of [35] refer to the “tribological layer” as a ratcheting layer (up to several microns thick), consisting of the polymer matrix reinforced with fractured SCFs, which was compacted (tamped) and polished due to repeated sliding of the counterface with an *Ra* surface roughness of 0.20–0.25 µm roughness. The tribological layer was formed from debris in two stages: a sharp increase in the CoF to a value of approximately ~0.4–0.5 followed by a subsequent decrease in CoF to about ~0.1–0.2. If the CoF level then remained stable, it can be asserted that the formed tribological layer was firmly adhered and fulfilled its functional role. In the following sections of this study, the interpretation of the results will be based on this assumption.

Loading PES with 10 wt.% SCFs 200 µm long reduced WR values by 9.6 times compared to that of neat PES, while enhancing the content of SCFs up to 30 wt.% was not accompanied by a further decrease. The reinforcement of PES with SCFs of 2 mm length in the composites PES/10SCF_2mm_ and PES/20SCF_2mm_ led to a significant reduction in both WR values and CoF (WR decreased by 11 and CoF by 2 times), whereas in PES/30SCF_2mm_, the wear intensity decreased only slightly (by 1.3 times compared to unfilled PES) with an average CoF of 0.47 (Table 3). Thus, it can be concluded that no tribological layer was formed on the wear track of the PES/30SCF_2mm_ composite.

**Figure 2:**
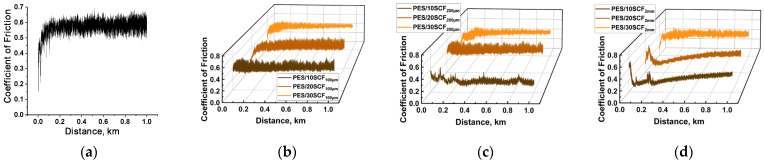
The CoF time dependencies for neat PES (**a**), as well as the PES/10-30SCF_100µm_ (**b**), PES/10-30SCF_200µm_ (**c**) and PES/10-30SCF_2mm_ (**d**) composites. The GCr15 steel counterface.

Figure 2 shows the time dependencies of the CoF for neat PES and all studied composites. For neat PES (Figure 2a), after a brief running-in period, the CoF maintained a consistently high value (~0.57) along with a high level of ‘high frequency’ (HF) oscillations. According to Figure 2b, the friction coefficient also had a high average value (0.411–0.503) and was characterized by a noticeable level of HF oscillation during the tribological test. According to the assumption made in this work, this indicates that no tribological layers had formed on the wear track surface.

**Figure 3:**
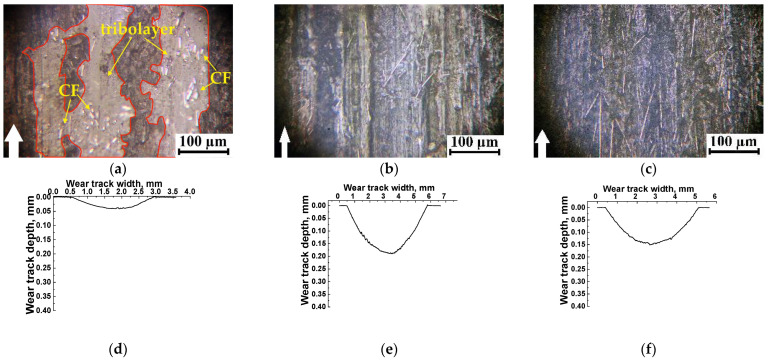
Optical micrographs of the wear track surfaces (**a**–**c**) and their profiles (**d**–**f**) on the PES/10SCF_200µm_ (**a**,**d**), PES/20SCF_200µm_ (**b**,**e**) and PES/30SCF_200µm_ (**c**,**f**) composites after the tribological tests. The GCr15 steel counterface. White arrows show the sliding direction.

A clear tendency for tribological layer formation was observed only in three composites, namely the PES/10SCF_200µm_ (Figure 2c and Figure 3a,d), PES/10SCF_2mm_ (Figure 2d) and PES/20SCF_2mm_ ones. Their CoF levels quickly reached a consistently low level of 0.20–0.25. In these cases, three stages were evident in the CoF time dependencies: (i) growth (running-in stage), (ii) reduction stage and (iii) stage of stable low-level (or gradually enhancing) CoF. For the PES/10SCF_200µm_ composite, the formed tribological layer, reinforced with fragmented (fractured) SCFs, is illustrated by the photo in Figure 3a (and its smooth wear track profile is presented in Figure 3d). Conversely, in the case of PES/20CF200 µm and PES/30CF200 µm, it is evident that the SCFs remained intact on the friction surfaces (Figure 3b,c), which did not allow the formation of a smooth (compacted) tribological layer. So, the wear track profiles were characterized by sawtooth shapes (Figure 3e,f).

According to the authors, when shorter SCFs of 200 μm length were loaded, the formation of a tribological layer was evident as a layer of mixture reinforced with fractured fibers, as illustrated by Figure 3a (as well as by a smooth tribotrack’s profile, Figure 3d). The regions outlined in red corresponded to the tribological layer. The latter was fragmented and non-uniform due to instability of adherence. This was manifested by the pattern of corresponding CoF time dependence (Figure 2c). When PES/20CF_200µm_ and PES/30CF_200µm_ composites were tested, the CF maintained their integrity (Figure 3b,c). The latter did not ensure the formation of a smooth (compacted) tribological layer, while the wear track possessed sawtooth profiles (Figure 3e,f).

The indicated stages are interpreted as follows: At the beginning of the tests (the running-in stage), a high and unstable CoF was observed. This can be explained by the interaction of asperities on the surface of the GCr15 steel counterface (Ra~0.25 µm) with those on the friction surface of the composite, formed as a result of grinding during sample preparation. A subsequent decrease in CoF to 0.1 occurred after a sliding distance of ~100 m. This is associated with the tribological layer formation and consolidation. A subsequent relatively constant CoF value of ~0.25 was observed at the test distance starting from 200 to 1000 m. The episodic instability of the CoF value may be related to the detachment and subsequent formation of new fragments of the tribological layers on the wear track surfaces. Although the process of tribolayer formation was initiated from the very beginning of the tests by adhered debris, the process was completed only at the test distances of about 100 m. This instability was explained by the high mutual activity of PES [3] and GCr15 steel components, whose interaction drives the development of tribo-oxidation reactions [36,37]. This phenomenon will be further discussed in the corresponding section.

In addition to the episodic short-term sharp increases in CoF values observed in the time dependencies (Figure 2c for the PES/10SCF_200µm_ composite, as an instance), a distinctive feature of such graphs during the formation of the tribolayer was the gradual increase in CoF during the third stage; this took place when PES was loaded with SCFs 2 mm length (Figure 2d for the PES/10SCF_2mm_ and PES/20SCF_2mm_ ones). It should be noted that some fractured SCFs 2 mm in length were evident. 

The authors believe that, as will be further investigated and discussed in the analysis of the topography of the wear track and other types of counterparts, the gradual increase in CoF with the formed tribolayer was due to more active interaction of the GCr15 steel counterpart and the composites, resulting in the shifting/flowing of the tribolayer relative to the bulk sample. Furthermore, when 2 mm long SCFs break down, their physical contacts with the bulk specimens are lost. The absence of such an effect in the composites with shorter SCFs (PES/10SCF_200µm_) was attributed by the authors to a more efficient retention (similar to tree roots) of the entire tribological layer, including due to a more stochastic directional distribution (compared to SCFs 2 mm long).

Since the ceramic counterpart is more inert with respect to the PES matrix, further tests were carried out in the ceramic–polymer tribological contacts under the same testing conditions for comparison purposes. It should be also noted that the use of the ceramic counterface was accompanied by two additional effects: its high hardness and lower heat dissipation. However, the role and contribution of these factors were not analyzed in detail within the framework of this study.

Table 5 presents the tribological characteristics of neat PES and PES-based composites obtained in tests against the ceramic counterface. From Table 5, it can be seen that the average CoF levels were approximately two times lower compared to those for the GCr15 steel counterface (Table 4). This result was evidently associated with the minimal development of tribo-oxidation processes in the tribological contact zone. At the same time, there was an increase in counterface temperature in the contact zone, which was likely due to the lower thermal conductivity of Al_2_O_3_. Replacing the GCr15 steel counterpart with the ceramic one reduced WR values by a factor of three for neat PES, although its CoF remained approximately at the same level. For the studied composites, a significant reduction in WR was observed across all aspect ratios, ranging from 7 to 43 times.

**Figure 4:**
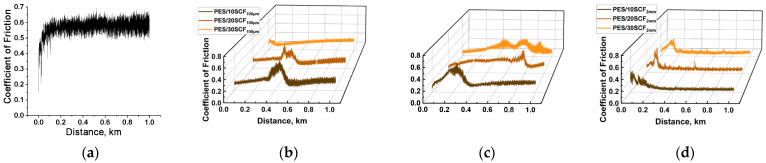
The CoF versus distance dependences for neat PES (**a**), as well as the PES/10-30SCF_100µm_ (**b**), PES/10-30SCF_200µm_ (**c**) and PES/10-30SCF_2mm_ (**d**) composites. The ceramic counterface.

In Figure 3, the dependencies of the CoF for all composites on the testing distance are presented. These results were also associated with the tribological layer formation. For neat PES (Figure 4a), the CoF level remained consistently high (~0.56) during the steady-state wear stage. After PES was loaded with SCFs 100 µm long at contents of 10 and 20 wt.% (Figure 3b,c), the running-in stage increased to 400 m, during which more intense wear of the composites occurred. However, the WR values did not exceed (2.3–3.6) × 10^−6^ mm^3^/N·m. At the same time, on the polished wear track surfaces, some fractured SCFs were seen (Figure 4a,b,d), which was one of the key criteria for the tribological layer formation. The observed local spikes in the CoF levels were likely related to damage and removal of the tribological layer fragments (during attempts at tribological layer formation and consolidation).

After PES was loaded with 30 wt.% SCFs 100 μm long, the running-in stage was short (within 100 m according to Figure 4d), accompanied by the lowest WR value of ~0.57 × 10^−6^ mm^3^/N·m. However, a gradual increase in the CoF level was recorded, similar to the cases shown in Figure 2d for the GCr15 steel counterface. This might indicate that SCFs 100 μm long were unable to prevent displacement (flow) of the formed tribological layer relative to the bulk sample. However, in terms of the achieved wear resistance for the PES/30SCF_100µm_ composite, the result was the highest. This could be attributed to the fact that fractured SCFs with minimal length are most effective in reinforcing the tribological layer.

For the PES/10SCF_200µm_ composite, the CoF changing kinetics was similar (Figure 4b), although shifted in distance compared to the cases with 10 and 20 wt.% SCFs 100 μm long (where the local peak corresponds to the tribological layer formation). Upon further increasing the contents of SCFs 200 μm long up to 20 and 30 wt.%, the trends of CoF changing kinetics were altered (Figure 3e). Although the average CoF levels over the entire test distance were within 0.2, it was not possible to conclude that the steady-state wear stage had been reached. As a result, the abrupt CoF changing kinetics was accompanied by WR values of ~(1.15–1.97) × 10^−6^ mm^3^/N m (which, however, are low values, particularly after PES was loaded with 30 wt.% SCFs 200 μm long).

**Figure 5:**
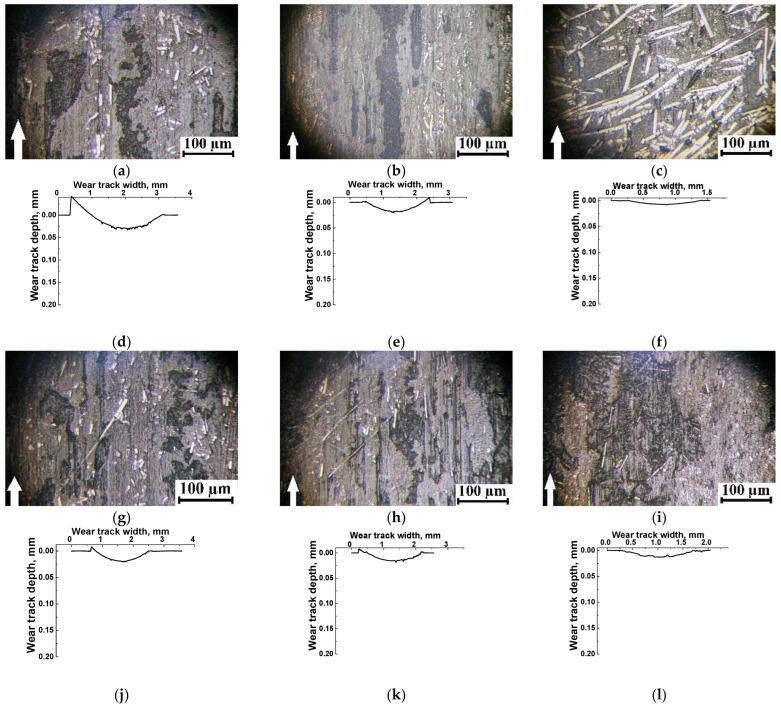
Optical micrographs of the wear track surfaces (**a**–**c**,**g**–**i**) and their profiles (**d**–**f**,**j**–**l**) on the PES/10SCF_100µm_ (**a**,**d**), PES/20SCF_100µm_ (**b**,**e**), PES/30SCF_100µm_ (**c**,**f**), PES/10SCF_200µm_ (**g**,**j**), PES/20SCF_200µm_ (**h**,**k**) and PES/30SCF_200µm_ (**i**,**l**) composites after the tribological tests. The ceramic counterface. White arrows show the sliding direction.

The photos presented in Figure 5g–i reveal that non-solid (fragmented) layers were formed on the wear track surfaces (as evidenced by their “torn” profiles, shown in Figure 5j–l). Only the PES/10SCF_200µm_ composite exhibited a distant reinforcement with fragmented SCFs, whose CoF changing kinetics followed the above-described interpretation of the conditions for the tribological layer formation.

Regarding the CoF composites with the longest SCF (2 mm long) (Figure 4d), the changing kinetics of CoF (a sharp increase followed by a gradual decrease to a stable level of 0.07–0.10) clearly indicated the formation of a continuous tribolayer. This means that the composition, fracture pattern of SCFs and the formed structure of the composites provided the ability to form, adhere and retain the wear-resistant tribological layers.

**Figure 6:**
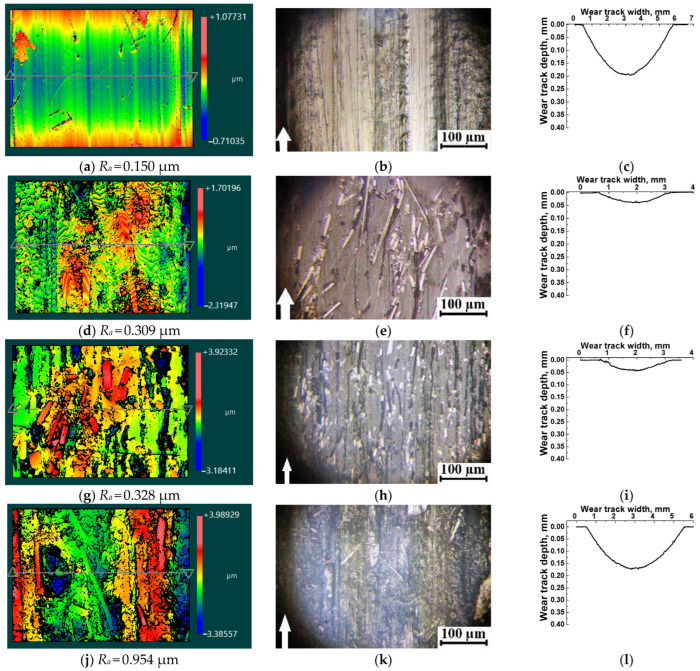
Optical profilometry images (**a**,**d**,**g**,**j**), micrographs (**b**,**e**,**h**,**k**) and stylus profilometry results on the wear tracks (**c**,**f**,**i**,**l**) after the tribological tests of neat PES (**a**–**c**), as well as the PES/10SCF_2mm_ (**d**–**f**), PES/20SCF_2mm_ (**g**–**i**) and PES/30SCF_2mm_ (**j**–**l**) composites. The GCr15 steel counterface. Image size of 140 μm × 105 μm (**a**,**d**,**g**,**j**). White arrows show the sliding direction.

**Figure 7:**
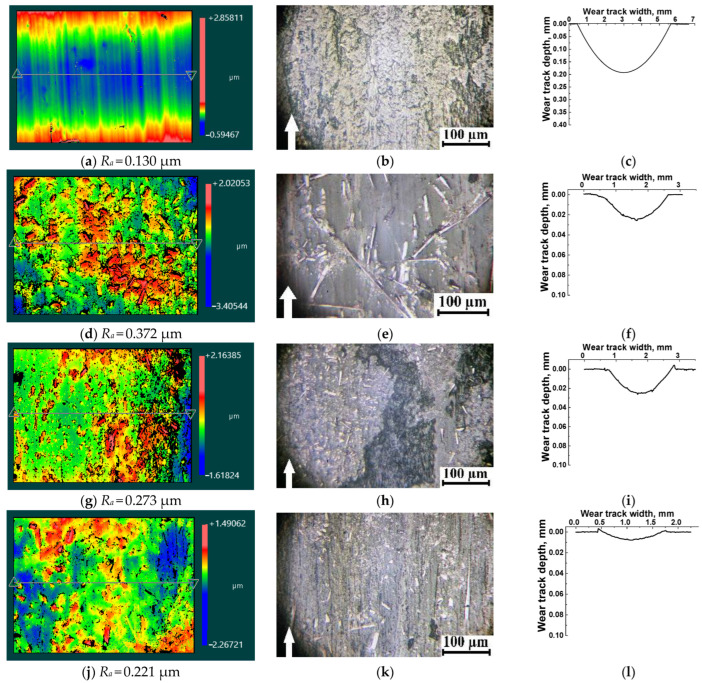
Optical profilometry images (**a**,**d**,**g**,**j**), micrographs (**b**,**e**,**h**,**k**) and stylus profilometry results on the wear tracks (**c**,**f**,**i**,**l**) after the tribological tests of neat PES (**a**–**c**), as well as the PES/10SCF_2mm_ (**d**–**f**), PES/20SCF_2mm_ (**g**–**i**) and PES/30SCF_2mm_ (**j**–**l**) composites. The ceramic counterface. Image size of 350 μm × 260 μm (**a**,**d**,**g**,**j**). White arrows show the sliding direction.

Since the composites loaded with SCFs 2 mm long showed higher tribological characteristics, their wear tracks were subjected to analysis. Figure 6 and Figure 7 show profilograms and micrographs of the wear track surfaces and their profiles on these specimens after the tribological tests against both GCr15 steel and ceramic counterfaces.

When tested against the GCr15 steel counterface, neat PES exhibited a high WR value, accompanied by the formation of longitudinal (relative to the sliding direction) grooves on the wear track surfaces, reaching up to 10 µm in width (Figure 6a–c).

In contrast, in the PES/10SCF_2mm_ and PES/20SCF_2mm_ composites, fragmented SCFs were observed on the wear surface, and the reduced wear intensity suggests the formation of fragmented (discontinuous) tribological layers (Figure 6e,h). The *Ra* surface roughness increased two-fold compared to neat PES (from 0.150 µm up to 0.328 µm). As seen in Figure 6f,i, the wear track depths in the composites decreased by an order of magnitude (compared to neat PES) due to the tribological layer formation.

In the PES/30SCF_2mm_ composite (Figure 6j–l), a tribological layer was not formed, although some adhered debris was observed on its wear track surface. In addition, no grinding signs were found there after the tribological test against the GCr15 steel counterface. Nevertheless, several furrows caused (Figure 6j) by the plowing action of the fractured SCFs were visible on the friction track. Consequently, the *Ra* surface roughness increased three-fold compared to the PES/20SCF_2mm_ composite (from 0.328 µm up to 0.954 µm).

When PES was tested against the ceramic counterface (Figure 7a–c), the temperature in the tribological contact increased up to 51 °C (Figure 6j) due to the low heat removal through Al_2_O_3_. This could have further stimulated the development of tribo-oxidation processes (which were accompanied by the formation of longitudinal furrows along the sliding direction (Figure 7a), as well as a high CoF level of ~0.56 and the oscillating pattern of its change (Figure 4a)) [35].

On the wear track surface of the PES/10SCF_2mm_ composite, some fractured SCFs were observed that enhanced the *Ra* surface roughness up to 0.372 µm (Figure 7d). In this case, a tribological layer was formed, which included both intact and fractured SCFs (Figure 7e). The partial preservation of intact SCFs could be caused by both their low quantity and activity in the development of mixing processes due to less active interaction with the ceramic counterface.

When the content of SCFs 2 mm long was increased to 20 wt.% (Figure 7g), the *Ra* surface roughness on the wear track decreased by ~35% (0.273 µm versus 0.372 µm), which corresponded to the formation of a discontinuous tribological layer reinforced with fragmented SCFs (Figure 7h). This was not accompanied by an increase in the CoF level, which remained extremely low (0.074), while the WR value, conversely, was quite high, WR = 3.77 × 10^−6^ mm^3^/N m. The authors attribute this to the discontinuous (non-solid) structure of the tribological layer.

A continuous tribological layer was observed only at the content of SCFs 2 mm long of 30 wt.% (Figure 7j,k). In this case, the *Ra* surface roughness decreased by 20%, and the WR value of 0.77 × 10^−6^ mm^3^/N m was minimal. However, it was slightly higher than in the case for the PES/30SCF_100μm_ composite (0.57 × 10^−6^ mm^3^/N m), although with a higher CoF level of ~0.183, as seen in Figure 5c,f. It should be noted that in the latter case, a large number of SCFs were visible on the wear track surface of the PES/30SCF_100μm_ composite, most of which were not fractured during friction. This indicates that SCFs 2 mm long could be part of the tribological layer. However, this effect developed only during friction against the ceramic counterpart, i.e., while minimizing chemical interaction between the components of the friction pair.

Based on the above, the formation of the wear-resistant tribological layer depended on the superposition of several factors, not only (i) the three-stage pattern of the CoF changing kinetics, reaching constant low values of 0.1–0.2 at the steady-state wear stage, but also (ii) low WR values, (iii) the formation of a polished continuous surface on the wear track and (iv) the reinforcement of PES with SCFs.

**Figure 8:**
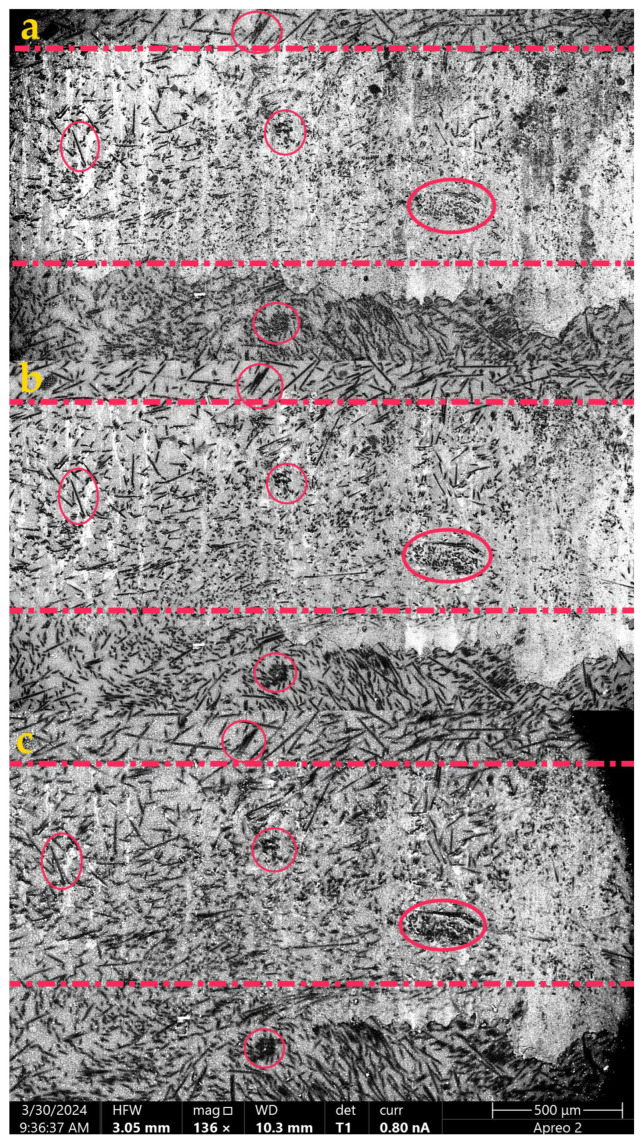
SEM micrographs of the wear track on the PES/30SCF_2mm_ composite after the tribological test against the ceramic counterface, obtained at accelerating voltages of 10 (**a**), 20 (**b**) and 30 (**c**) kV. Red dashed lines contour the wear tracks. Red circles mark the agglomeration of SCFs.

A more detailed analysis of the surface topography of the wear track of the PES/30SCF_2mm_ composite in terms of tribological layer formation was carried out. For this purpose, the structure of both tribological and subsurface layers in the PES/30 CF2mm composite during friction against a ceramic counterface was investigated using SEM at different accelerating voltages (10, 20 and 30 kV). It is known that changing the accelerating voltage value enables the penetration depth of electrons into a substance to be varied. Consequently, the backscattered electrons from such depths reflected patterns shown in Figure 8. X-ray computed micro-CT can provide more detailed information on the tribological structure [38,39]. However, the thickness of the latter in units of microns might be too small to be duly resolved by micro-CT means.

At an accelerating voltage of 10 kV (enabling the investigation of the wear track surface), in addition to some adhered fragments of debris, a large number of fractured SCFs was observed (Figure 8a). At 20 kV (Figure 8b), the wear track boundaries became less defined, and the number of visible fragments of debris decreased. At an accelerating voltage of 30 kV (Figure 8c), which allowed us to examine the subsurface layer, intact SCFs were visible, remaining undamaged during the tribological test. Thus, it can be “roughly” assumed that the thickness of the tribological layer was limited by this depth (as a distance from the wear track surface).

**Figure 9:**
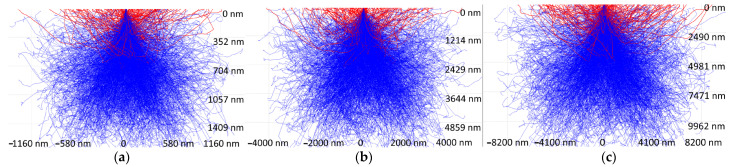
The X-ray (blue lines) and backscattered electron (red lines) plumes in neat PES, obtained at accelerating voltages of 10 (**a**), 20 (**b**) and 30 (**c**) kV.

To assess the depth of electron beam penetration, the methodology described in [40] was employed. The corresponding backscattered electron and X-ray plumes were calculated using the “Casino” (CASINO_v2.4.8.1) free software, based on the Monte Carlo method [40]. As shown in Figure 9, electrons were backscattered at a depth of 610 nm relative to the surface for U = 10 kV, while for U = 20 kV and U = 30 kV, this depth was approximately 1850 nm and 3500 nm, respectively. For this reason, this approach allowed us to study the only thin subsurface layer, whose structure inevitably changed upon testing. Based on the obtained data, it could be concluded that the tribological layer thickness was approximately ~2–4 µm for the PES/30SCF_2mm_ composite. On the other hand, another estimate of the tribological layer thickness could be obtained based on the characteristic diameter of SCFs, by which the tribological layer was actually reinforced. In this case, its thickness could reach 6–7 µm.

**Figure 10:**
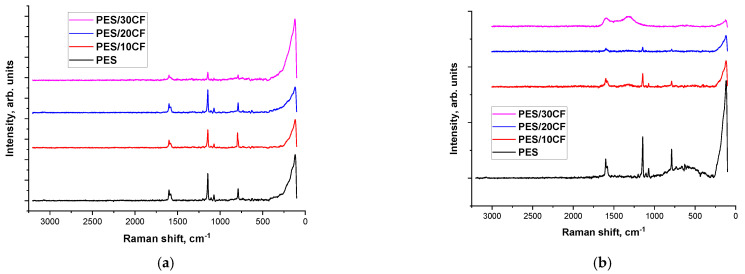

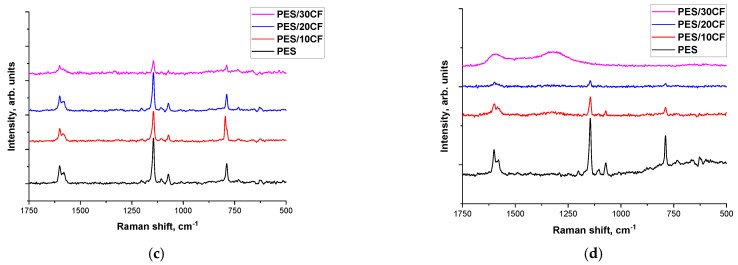
The Raman spectra obtained for neat PES and the composites loaded with SCFs 2 mm long (**a**,**c**) and for their wear tracks (**b**,**d**) before (**a**,**c**) and after (**b**,**d**) the tribological tests. The GCr15 steel counterface.

Figure 10 shows Raman spectra of neat PES and the composites loaded with SCFs 2 mm long before and after the tribological tests against the GCr15 steel counterface. For the non-tested samples, both neat PES and the composites (Figure 10a,c) exhibited characteristic peaks from sulfones and sulfoxides (SO_2_—1000–1420 cm^−1^; S=O—1225–980 cm^−1^; S–O—870–690 cm^−1^) and C–S—570–710 cm^−1^. In the 1610–1790 cm^−1^ region of reciprocate wavelengths, characteristic peaks corresponding to ethers (C=C and C–C) were observed. At the same time, the Raman spectra within the wear tracks (Figure 10b,d) indicated the development of tribochemical processes during the tribological testing. Changes in the spectrum of neat PES were observed in the 570–710 cm^−1^ region of reciprocate wavelengths. Specifically, as the content of SCFs increased, the peaks from sulfones and sulfides, as well as from ether compounds, decreased down to zero. This suggested a change in the chemical composition of the wear track surface layer: in the spectrum of the PES/30SCF_2mm_ composite, two diffuse maxima were observed in the 1650 and 1350 cm^−1^ range, indicating the formation of more complex esters and oxides.

**Figure 11:**
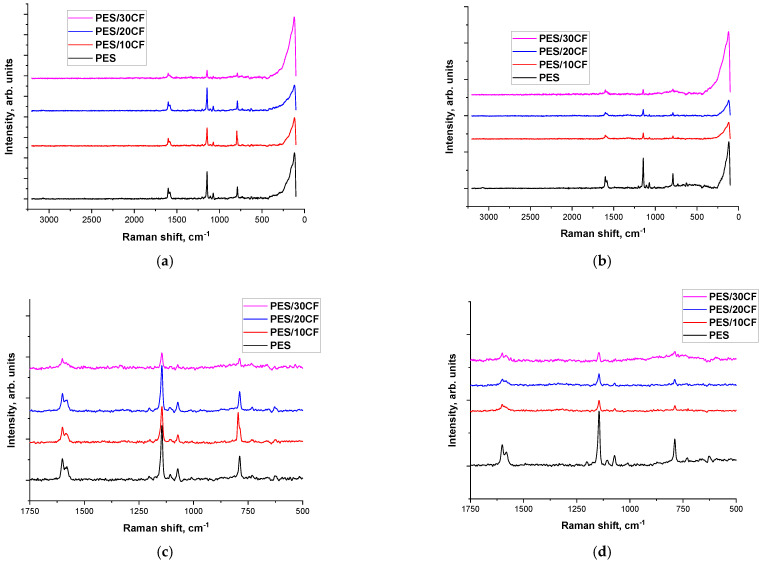
The Raman spectra obtained for neat PES and the composites loaded with SCFs 2 mm long (**a**,**c**) and for their wear tracks (**b**,**d**) before (**a**,**c**) and after (**b**,**d**) the tribological tests. The ceramic counterface.

A quite different pattern was revealed for the ceramic–polymer tribological contact (Figure 11). For the wear track of neat PES (Figure 11c), the Raman spectrum was identical to the initial one (Figure 11a). For the composites with increasing the content of SCFs, the amplitude of the peaks from ether and sulfonic compounds decreased. However, the chemical structure did not change: within the wear track, the intensity of the peaks was weaker (Figure 11b,d) compared to the initial ones (Figure 11a,c).

Therefore, the fact that tribo-oxidation processes occurred during the tests against the GCr15 steel counterface suggested that the latter interacted more actively (including in terms of shear strains in both tribological and subsurface layers) with the studied composites.

Since metal–polymer contacts are significantly more common than ceramic–polymer ones, the obtained results are discussed below as a comparison with those for the less-“active” AISI321 steel counterpart.

## 4. Discussion

A key result was the identification and characterization of the role of the tribological layer in the wear resistance of PES-based composites in linear tribological contacts. However, concurrently, an interesting finding was revealed regarding the two-fold higher WR values of PES/SCF composites during testing against the GCr15 steel counterface (due to its greater chemical activity) than those for the ceramic one. In this regard, it was decided to conduct additional studies of the tribological characteristics of the composites loaded with SCFs 2 mm long during testing against the AISI321 steel counterpart. Table 6 illustrates the results. As can be seen from this table, the average CoF levels were approximately twice as high as those for the GCr15 steel counterpart (Table 3). Regarding the WR values, upon replacing the GCr15 steel counterpart with the AISI321 one, this value increased by ~1.5 times at the contents of SCFs of 10 and 20 wt.%, while for 30 wt.%, the WR value, on the contrary, decreased by almost an order of magnitude (down to 4.4 × 10^−6^ mm^3^/N m). It should be recalled that during the friction of the latter composite against GCr15 steel, a tribological layer was not formed, and the WR value was high, WR = 48.4 × 10^–6^ mm^3^/N·m). 

**Figure 12:**
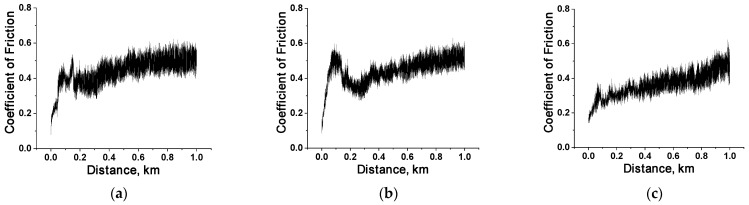
The CoF versus distance dependences for the PES/10SCF_2mm_ (**a**), PES/20SCF_2mm_ (**b**) and PES/30SCF_2mm_ (**c**) composites. The AISI321 steel counterface.

**Figure 13:**
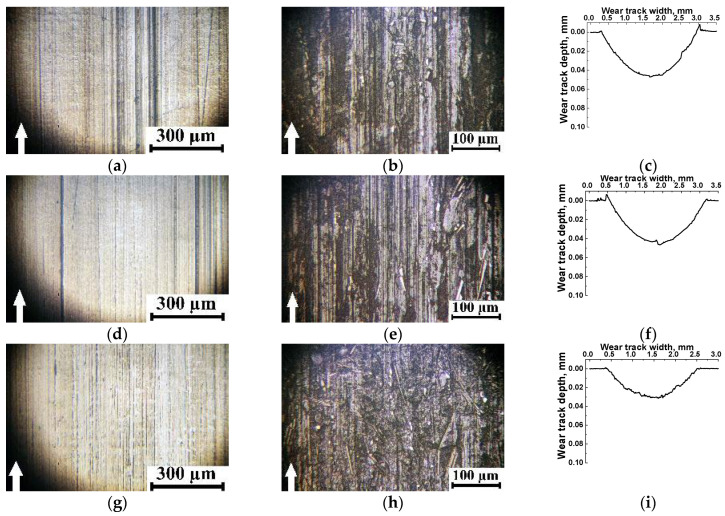
The optical micrographs of the surface on the AISI 321 steel counterface (**a**,**d**,**g**), as well as wear track surfaces (**b**,**e**,**h**) and their profiles (**c**,**f**,**i**), after the tribological tests of the PES/10SCF_2mm_ (**a**–**c**), PES/20SCF_2mm_ (**d**–**f**) and PES/30SCF_2mm_ (**g**–**i**) composites. White arrows show the sliding direction.

The authors believe that the increase in WR values to ~(7.9–8.2) ×10^−6^ mm^3^/N m for the tested composites was attributed to the absence of tribological layers (Figure 13b,e). CoF levels reached ~0.40–0.45 almost immediately and remained relatively constant thereafter (Figure 12a,b). For the PES/30SCF_2mm_ composite, the CoF level was lower (~0.25) at the end of the running-in stage (at a testing distance of about 100 m), but it gradually increased then (Figure 12c). This pattern was to some extent consistent with the results of the tribological tests against the GCr15 steel counterpart, which can be interpreted as the flow/displacement of the (not fully formed) tribological layers relative to the bulk specimens (Figure 2d for the PES/10SCF_2mm_ and PES/20SCF_2mm_ composites, respectively). The WR values were comparable: WR = 4.4 × 10^−6^ mm^3^/N m for the tribological contact of the PES/30SCF_2mm_ composite with the AISI 321 steel counterface and WR = 5.9–6.1 × 10^−6^ mm^3^/N m for the composites for the lower contents of SCFs 2 mm long with the GCr15 one.

Figure 13 shows the optical micrographs of the surface on the AISI 321 steel counterface, as well as wear track surfaces and their profiles after the tribological tests of the PES/10SCF_2mm_, PES/20SCF_2mm_ and PES/30SCF_2mm_ composites. For the PES/10 SCF_2mm_ one, scratches and grooves 10–15 µm wide were evident on the surfaces of both tribological contact components (Figure 13a–c). Their formation was caused by the damaging (scratching) effect of reinforcing SCFs, at least in part because the stainless austenitic steel was characterized by lower hardness compared to that of the GCr15 bearing grade.

Increasing the content of SCFs 2 mm long up to 20 wt.% reduced the number of large scratches on the surface of the AISI321 steel counterface (Figure 13d), while the wear track surface topography (Figure 13e) was similar to that for the PES/10SCF_2mm_ composite (Figure 13b). Since the WR values for them were close, it can be assumed that damage to the surface of the AISI321 steel counterpart did not significantly affect the interaction of the rubbing components in the first case as well.

It should be emphasized once again that the use of the AISI 321 steel did not ensure the formation of the tribological layers on the studied PES-based composites. At the steady wear stage, there was a flow of the surface layers of the polymer matrix material in the sliding direction (Figure 13c,f), where the steel ring was in contact with the composite blocks upon testing. This phenomenon explains the gradual increase in the CoF levels with an increase in the tribological test distance (Figure 12a,b).

For the PES/30SCF_2mm_ composite, grinding, during friction, was observed not only on the surfaces on the AISI321 steel counterface (Figure 13g) but also on the wear track (Figure 13h). This resulted in a decrease in the WR value by almost two times; however, the fact of the tribological layer formation according to the criteria proposed above was not fully confirmed. 

Nevertheless, if we rank all three types of counterfaces used in terms of their effectiveness in forming tribological properties in PES/30SCF_2mm_ composites, ceramic Al_2_O_3_ was the most effective, while the GCr15 was the least. The authors believe that this was primarily due to their different chemical activity relative to the PES matrix.

The lower activity of AISI321 austenitic steel (compared to the GCr15 bearing grade) resulted in a lower CoF level, especially in the initial stage of the tribological testing (Figure 12c). However, both steel counterfaces did not eliminate wear and suppressed the possibility of forming an appropriate tribological layer (Figure 13h). This task was even more difficult to solve in the case of sliding against the GCr15 steel counterface, when the CoF level of 0.47 was initially high; therefore, no tribological layer was formed. On the other hand, the WR value was initially extremely low upon testing against the least active ceramic counterface; therefore, the tribological layer formation was achieved on the wear track layer at a shallow depth (apparently, not exceeding the diameter of the SCFs). In this case, minimizing polymer wear on a well-run-in sliding surface of PES/30SCF2mm locally destroying the SCFs and compacting the tribolayer was a more easily solvable task. 

**Figure 14:**
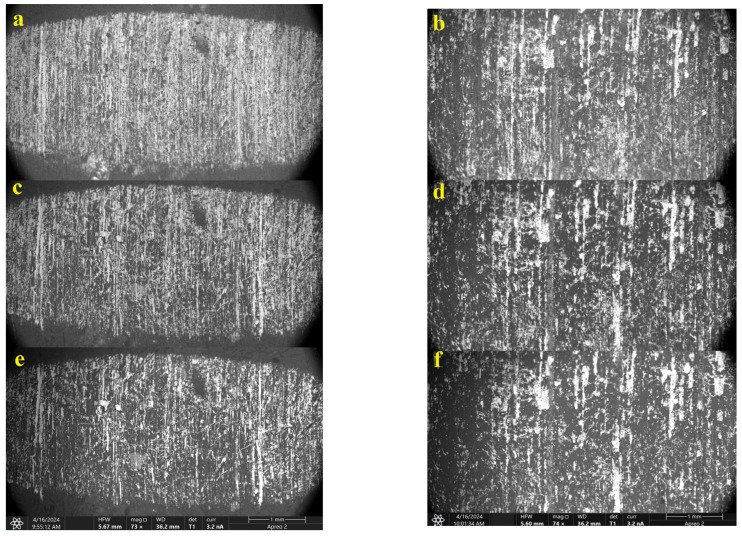
The SEM micrographs of the wear track surfaces on the PES/30SCF_2mm_ composite after the tribological tests against the AISI321 (**a**,**c**,**e**) and GCr15 (**b**,**d**,**f**) steel counterfaces. Accelerating voltages of 10 (**a**,**b**), 20 (**c**,**d**) and 30 (**e**,**f**) kV.

To compare the structures of the subsurface layers on the wear tracks of the PES/30SCF_2mm_ composite after the tribological tests against both steel counterfaces, their SEM examinations were carried out at different accelerating voltages (Figure 14). It can be concluded from the obtained data that the wear track surface was smoother after testing against the stainless steel counterface (Figure 14a). As the depth of electron penetration increased, the topography pattern changed (Figure 14c,e): reinforcing SCFs were visible, which corresponded to the structure of the bulk specimen. After testing against the bearing steel counterpart, the topography of the wear track surface was characterized by the absence of a tribological layer (Figure 14b,d,f) that reflected the WR value of ~48.4 × 10^−6^ mm^3^/N m. At all penetration depths of electrons, the SEM micrographs were similar. This, under conditions of intensive wear, was partly due to the presence of the deep and wide (approximately 50 µm) grooves shown above (Figure 6j,k) oriented along the sliding direction of the counterface. Increasing the accelerating voltage enabled the examination of the structure at depths up to 2 μm. This did not allow differences between the surface layer and the bulk composite to be revealed (this task was solved after the tests of the PES/30SCF_2mm_ composite against two other types of counterfaces under conditions of tribological layer formation).

**Figure 15:**
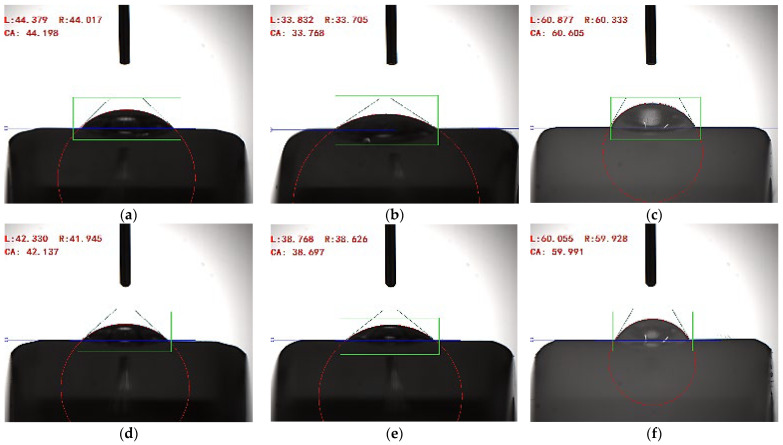
The contact wetting angles of drops on the GCr15 steel (**a**,**d**), 321 steel (**b**,**e**) and ceramic (**c**,**f**) counterfaces. Distilled water (**a**–**c**), ethylene glycol (**d**–**f**).

To quantitatively characterize the different activities of the counterfaces, contact wetting angle measurements were performed using distilled (deionized) water and ethylene glycol on the counterface surfaces using an “LR-SDC-100” optical contact angle tester (Dongguan Lonroy Equipment Co., Ltd., Dongguan, China). For this purpose, 3 μL drops of both liquids were carefully placed on each counterface with a microsyringe. Optical images were taken 1 s after the water drop landed on the counterface surface. The contact wetting angles (Figure 14) were measured using the software integrated into the tester (water drop angle measuring instrument, ISO15989). For both distilled water (the most polar liquid) and ethylene glycol (less polar and more viscous), the maximum contact wetting angle was ~60° on the ceramic counterface (Figure 15c,f). The lowest values of 33.7°/38.7° were determined on the AISI 321 steel one (Figure 15b,e), while they were slightly higher at 44.2°/42.1° on the GCr15 one (Figure 15a,d). The ranking results based on the contact wetting angles differed slightly from those based on tribological characteristics. This may be partially due to the fact that not only the activity of the counterpart materials affected their functional properties.

The authors acknowledge that for accurate measurement of surface energy, the value of which was proportional to the activities of the studied materials, it is necessary to use a set of liquids (at least four types) with different degrees of polarity [41]. In addition, the contact wetting angles were determined on the rounded surfaces, which can also affect the measurement results. Nevertheless, since the task was rather to qualitatively characterize the difference between the steels and the ceramic, the obtained data can be considered quite informative.

It should be also noted that SCFs 100 μm long were viscose, i.e., they possessed lower strength properties. However, with their content of 30 wt.%, the wear resistance of the studied composites was also improved in the ceramic–polymer tribological contacts through the tribological layer formation (this aspect was not specifically discussed in the work).

Note that the mechanism of tribological layer formation was reported in the literature as a combination of ratcheting and mixing of components. In classical terms, this meant adhesion and deformation mechanisms of wear. In tribological layer formation, these mechanisms were dominant. However, the damaging effect of scratching CFs could not be excluded. Thus, micro-abrasion wear by CFs was additionally involved when no tribological layer able to protect the sliding surface of the polymer matrix composite had been formed. 

**Figure 16:**
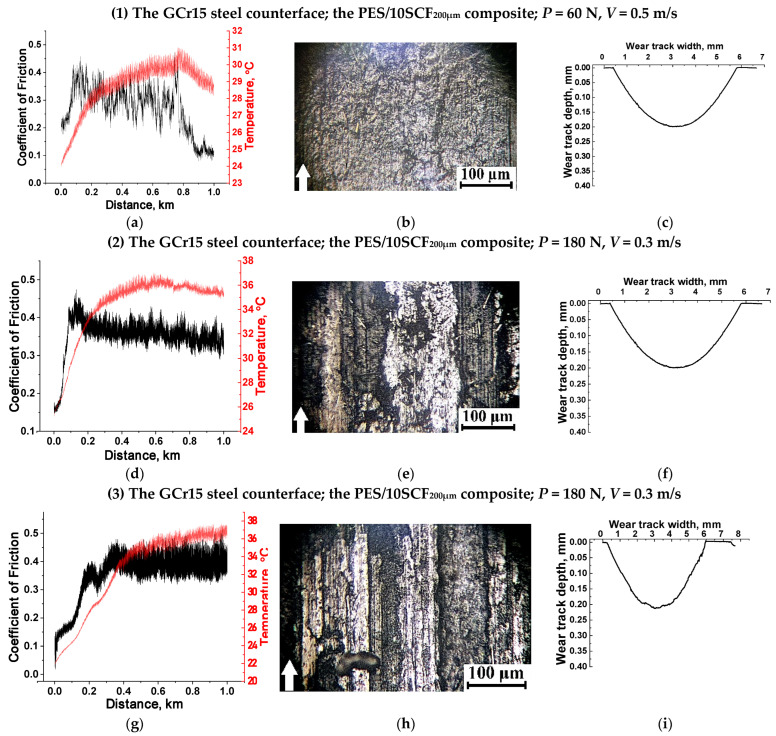

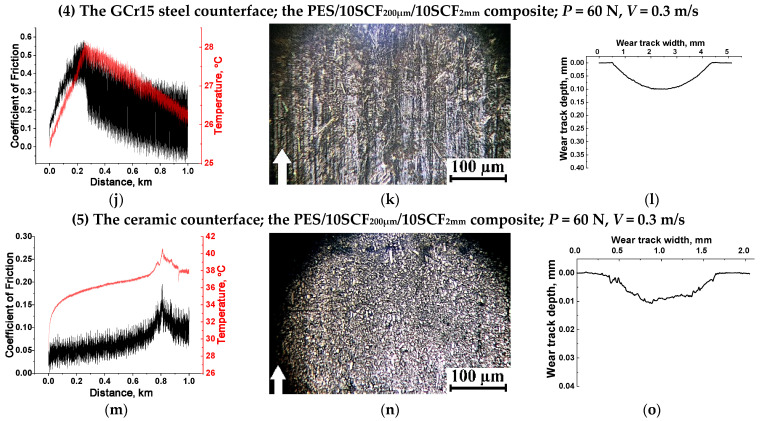
The CoF versus distance and temperature versus distance dependencies (**a**,**d**,**g**,**j**,**m**) and the optical micrographs of the wear track surfaces (**b**,**e**,**h**,**k**,**n**) and their profiles (**c**,**f**,**i**,**l**,**o**) after the tribological tests of the PES/10SCF_200μm_ (**a**–**f**), PES/10SCF_2mm_ (**g**–**i**) and PES/10SCF_200μm_/10SCF_2mm_ (**j**–**o**) composites. GCr15 steel (**a**–**l**) and ceramic (**m**–**o**) counterfaces. White arrows show the sliding direction.

As further confirmation of the determining role of tribological layers in the investigated characteristics, the results of some additional tests are presented in Table 7 and Figure 16. In these cases, the test conditions did not contribute to the tribological layer formation. The authors do not provide a detailed description of them; it is sufficient to indicate what conditions led to these changes (discussed below).

No. 1. For the PES/10SCF_200μm_ composite, an increase in the *V* sliding speed up to 0.5 m/s at the *P* load of 60 N enhanced both the temperature of the GCr15 steel counterface up to 31.0 °C and WR value up to ~15.8 × 10^−6^ mm^3^/N·m but maintained a low and unstable CoF level of ~0.205 (Figure 16a–c).

Nos. 2 and 3. For both PES/10SCF_200μm_ and PES/10SCF_2mm_ composites (Figure 16d–f and g–i, respectively), enhancing the *P* load up to 180 N at a *V* sliding speed of 0.3 m/s was accompanied by a noticeable increase in WR values up to ~(25.6–26.6) × 10^−6^ mm^3^/N·m due to high CoF levels of ~0.35–0.39 and rising temperature of the GCr15 steel counterface up to 37 °C.

Nos. 4 and 5. A three-component PES/10SCF_200μm_/10SCF_2mm_ composite was additionally fabricated containing 10 wt.% SCFs of both lengths 200 µm and 2 mm. It was tested under the typical tribological conditions (*P* = 60 N, *V* = 0.3 m/s). Upon testing against the GCr15 steel counterface, a discontinuous tribological layer was formed in the final stage (Figure 16k). However, this was preceded by an increase in the CoF level up to 0.5 followed by its long-term decrease at extremely high HF oscillations (Figure 16j), which was accompanied by a high level of WR ~27.4 × 10^−6^ mm^3^/N·m (Figure 16l), which, however, was practically identical to the above-described PES/10SCF_200μm_ and PES/10SCF_2mm_ composites (Nos. 2 and 3). Testing against the ceramic counterface enhanced its temperature up to 40.3 °C. Nevertheless, a typical tribological layer was formed at a CoF level of ~0.1 and a WR value of ~0.93 × 10^−6^ mm^3^/N·m (Figure 16m–o). Such patterns correlated well with those for the PES/30SCF_2mm_ composite (CoF~0.08 and WR~0.8 × 10^−6^ mm^3^/N·m according to Figure 7k,l).

Table S1 (Supplementary) also presents the results of tribological tests of other three-component PES-based composites reinforced with SCFs and additionally loaded with particles of different natures and sizes, including nanosized ones [42]. In the vast majority of cases, WR values of >50 × 10^−6^ mm^3^/N·m were comparable to those of neat PES, since no tribological layers occurred in this case. Thus, tribological layer formation is an effective mechanism for improving the wear resistance of non-antifriction PES-based composites. Nevertheless, more severe load–speed tribological conditions can eliminate tribological layer formation, deteriorating the tribological characteristics.

## 5. Conclusions

The tribological characteristics of the PES-based composites reinforced with SCFs with aspect ratios of 14–250 and the contents of 10–30 wt.% were investigated for linear metal–polymer and ceramic–polymer contacts according to the “block-on-ring” scheme. It was shown that even when using (non-antifriction) polymer PES as a matrix material with a high friction coefficient, a significant increase in wear resistance could be achieved by implementing a tribolayer formation mechanism. By “tribological layer”, the authors mean a layer of ratcheting up to several microns thick, based on a polymer matrix, reinforced with fragmented SCFs, compacted and polished by repeated sliding against a rough counterpart. According to SEM micrographs, the tribological layer thicknesses can be 2–7 µm, where the upper limit is determined by the diameter of the SCFs loaded to reinforce the PES-based composites.

Several conditions are proposed according to which a tribological layer (in fact, from debris) was formed under linear tribocontact, including the three-stage pattern of the changing kinetics of CoF levels. They sharply increased up to ~0.4–0.5 in the first (running-in) stage and gradually decreased down to ~0.1–0.2 in the second stage. Then, if the CoF levels did not change, it can be argued that the formed tribological layer had become fixed and performed its functional role. In addition, the following patterns have been identified:

1. Upon testing against the GCr15 steel counterface, as the most active in relation to PES, the tribological layers were formed only on the wear tracks of the PES/10SCF_200µm_, PES/10SCF_2mm_ and PES/20SCF_2mm_ composites. After PES was loaded with SCFs 2 mm long, the distinctive feature of the CoF versus distance dependences was its gradual increase in the third wear stage due to the displacement/flow of the tribological layers relative to the bulk specimens. Shorter SCFs were more effective at retaining the tribological layer, since they, like tree roots, penetrated without fracturing to significant depths from the surface of the PES/10SCF_200µm_ composite.
2. In the ceramic–polymer tribological contacts, the average CoF levels decreased by multiples, primarily due to the lower activity of the ceramic counterface compared to the GCr15 steel one. At the same time, WR values also reduced significantly, and the wear track surfaces were smooth. The PES-based composites loaded with SCFs 2 mm long were characterized by the minimum CoF levels, for which their three-stage changing pattern corresponded to one of the conditions for tribological layer formation. However, only loading PES with 30 wt.% SCFs provided the minimum WR value of 0.77 × 10^−6^ mm^3^/N m. An important condition for tribological layer formation was the localization of deformation processes (shear strains and mixing with fragmented SCFs) only in a thin subsurface layer on the wear track surfaces.
3. During the tribological test of the PES/30SCF_2mm_ composite against the AISI321 steel counterface, “intermediate” in terms of activity, the WR value was reduced to 4.4 × 10^−6^ mm^3^/N m (by an order of magnitude compared to the GCr15 bearing grade). Nevertheless, it was not possible to form an effective tribological layer (reinforced with fractured SCFs) similar to sliding against the ceramic counterface.
4. More severe load–speed tribological conditions prevented the formation of a tribological layer when the PES/10SCF_200µm_ and PES/10SCF_2mm_ composites were tested against the GCr15 steel counterface, which was accompanied by an increase in WR values by 2–3 times relative to *P* = 60 N and *V* = 0.3 m/s.
5. In the ceramic–polymer tribological contact at *P* = 60 N and *V* = 0.3 m/s, an effective tribological layer was formed on the wear track surface of the three-component PES-based composite containing 10 wt.% SCFs of lengths 200 µm and 2 mm, so its tribological characteristics were identical to those of the PES/30SCF_2mm_ sample.

## Figures and Tables

**Table 1:** Material properties of commercial PES in use.

Properties	Typical Value Unit
Physical	
Density/Specific Gravity	1.37
Melt Mass-Flow Rate (MFR) (380 °C/2.16 kg)	10 to 13 g/10 min
Molding Shrinkage—Flow	0.60%
Water Absorption (24 h)	0.56%
Thermal	
Deflection Temperature Under Load 1.8 MPa, Annealed	200 °C
Melting Temperature	225 °C
Maximum Service Temperature	180 °C
Mechanical	
Elastic Modulus *E*	2600 MPa
Ultimate Tensile Strength σ_UTS_	91.0 MPa
Elongation at Break ε	>50%

**Table 2:** The fillers for the PES-based composites.

Brand, Manufacturer	Precursor	Length, µm	Aspect Ratio (AR)	Elastic Modulus, GPa	Ultimate Tensile Strength (UTS), MPa	Composite Designation
UVI-12, MPRI Belarus NAS, Gomel, Belarus	Viscose	100	14	60	1200	PES/10SCF_100µm_
Tenax^®^-A, Teijin Carbon Europe Gmbh, Heinsberg, Germany	PAN	200	28	200	2600	PES/10SCF_200µm_
Tenax^®^-A, Teijin Carbon Europe Gmbh, Heinsberg, Germany	PAN	2000	250	200	2600	PES/10SCF_2mm_

**Table 3:** The physical and mechanical properties of neat PES and PES-based composites.

No.	Composite	Density *ρ*, (g/cm^3^)	Shore *D* Hardness	Elastic Modulus *E* (GPa)	Ultimate Tensile Strength σ_UTS_ (MPa)	Elongation at Break ε (%)
1	Neat PES	1.40	77.2 ± 1.3	2.57 ± 0.06	85.4 ± 2.1	37.90 ± 4.80
2	PES/10SCF_100µm_	1.39	78.7 ± 0.3	3.32 ± 0.11	94.1 ± 0.3	5.44 ± 0.55
3	PES/20SCF_100µm_	1.40	78.9 ± 1.1	4.07 ± 0.32	85.7 ± 0.3	2.62 ± 0.16
4	PES/30SCF_100µm_	1.48	83.1 ± 0.7	6.13 ± 0.34	50.6 ± 1.8	0.87 ± 0.04
5	PES/10SCF_200µm_	1.45	80.4 ± 0.9	7.08 ± 0.34	117.9 ± 3.4	2.24 ± 0.12
6	PES/20SCF_200µm_	1.41	80.0 ± 0.5	6.26 ± 0.10	121.7 ± 2.8	2.64 ± 0.12
7	PES/30SCF_200µm_	1.49	83.2 ± 1.3	13.76 ± 0.24	153.1 ± 8.3	1.36 ± 0.08
8	PES/10SCF_2mm_	1.44	80.1 ± 1.0	7.63 ± 0.31	108.5 ± 7.9	1.49 ± 0.09
9	PES/20SCF_2mm_	1.42	80.0 ± 0.5	7.18 ± 0.18	107.0 ± 9.0	1.56 ± 0.16
10	PES/30SCF_2mm_	1.44	84.2 ± 0.5	16.8 ± 0.70	136.0 ± 8.0	0.86 ± 0.07

**Table 4:** The tribological properties of neat PES and the PES-based composites. The GCr15 steel counterface.

No.	Composite	CoF	WR, mm^3^/N·m, 10^−6^	Temperature, °C
0	Neat PES	0.577 ± 0.037	66.7 ± 2.65	32.1 ± 1.2
1	PES/10SCF_100µm_	0.411 ± 0.050	63.2 ± 1.2	27.5 ± 0.6
2	PES/20SCF_100µm_	0.452 ± 0.057	73.6 ± 1.1	26.4 ± 1.2
3	PES/30SCF_100µm_	0.503 ± 0.025	61.6 ± 3.2	25.5 ± 1.5
4	PES/10SCF_200µm_	0.197 ± 0.033	6.9 ± 1.4	25.8 ± 1.3
5	PES/20SCF_200µm_	0.421 ± 0.051	69.3 ± 4.7	24.9 ± 1.3
7	PES/30SCF_200µm_	0.450 ± 0.034	59.2 ± 0.8	25.7 ± 1.2
8	PES/10SCF_2mm_	0.259 ± 0.043	6.1 ± 1.0	27.2 ± 0.5
9	PES/20SCF_2mm_	0.307 ± 0.053	5.9 ± 1.2	24.1 ± 1.0
10	PES/30SCF_2mm_	0.472 ± 0.025	48.4 ± 1.6	24.8 ± 1.5

**Table 5:** The tribological characteristics of neat PES and PES-based composites. The ceramic counterface.

No.	Composite	CoF	WR, mm^3^/N·m, 10^−6^	Temperature, °C
0	Neat PES	0.560 ± 0.035	24.62 ± 1.36	51.0 ± 2.1
1	PES/10SCF_100µm_	0.162 ± 0.031	3.60 ± 0.51	51.5 ± 2.8
2	PES/20SCF_100µm_	0.141 ± 0.030	2.32 ± 0.17	44.3 ± 0.4
3	PES/30SCF_100µm_	0.183 ± 0.028	0.57 ± 0.06	38.2 ± 3.2
4	PES/10SCF_200µm_	0.161 ± 0.020	2.30 ± 0.16	49.4 ± 3.5
5	PES/20SCF_200µm_	0.197 ± 0.061	1.97 ± 0.05	46.3 ± 0.6
7	PES/30SCF_200µm_	0.173 ± 0.059	1.15 ± 0.21	48.2 ± 2.7
8	PES/10SCF_2mm_	0.059 ± 0.011	3.78 ± 0.34	30.3 ± 0.9
9	PES/20SCF_2mm_	0.074 ± 0.013	3.77 ± 0.29	30.8 ± 0.6
10	PES/30SCF_2mm_	0.077 ± 0.017	0.77 ± 0.08	36.8 ± 1.4

**Table 6:** The tribological characteristics of the PES-based composites. The 321 steel counterface.

No.	Composite	CoF	WR, mm^3^/N·m, 10^−6^	Temperature, °C
1	PES/10SCF_2mm_	0.459 ± 0.055	8.15 ± 0.35	26.75 ± 1.30
2	PES/20SCF_2mm_	0.466 ± 0.046	7.90 ± 0.61	26.51 ± 1.11
3	PES/30SCF_2mm_	0.379 ± 0.054	4.43 ± 0.19	25.43 ± 0.99

**Table 7:** The additional tribological characteristics of the PES-based composites. The “block-on-ring” scheme; test distance of 1 km.

No.	Composite	CoF	WR, mm^3^/N·m, 10^−6^	Temperature, °C
The GCr15 steel counterface; *P* = 60 N *V* = 0.5 m/s				
1	PES/10SCF_200μm_	0.205 ± 0.079	15.77 ± 2.23	31.0 ± 1.1
The GCr15 steel counterface; *P* = 180 N *V* = 0.3 m/s				
2	PES/10SCF_200μm_	0.348 ± 0.018	26.61 ± 0.21	37.0 ± 2.0
3	PES/10SCF_2mm_	0.386 ± 0.035	25.57 ± 0.91	37.7 ± 4.0
The GCr15 steel counterface; *P* = 60 N *V* = 0.3 m/s				
4	PES/10SCF_200μm_/10SCF_2mm_	0.189 ± 0.126	27.38 ± 0.19	28.2 ± 0.5
The ceramic counterface; *P* = 60 N *V* = 0.3 m/s				
5	PES/10SCF_200μm_/10SCF_2mm_	0.099 ± 0.014	0.93 ± 0.07	40.6 ± 1.3

## Data Availability

Data is contained within the article or Supplementary Materials.

## Supplementary Materials

The following supporting information can be downloaded at: https://www.mdpi.com/article/10.3390/polym16152180/s1, Table S1: The results of the tribological tests of the PES-based composites.
