# Supplementary material for: The Role of Triboloading Conditions in Tribolayer Formation and Wear Resistance of PES-Based Composites Reinforced with Carbon Fibers
Source: Polymers (Basel). 2024 Jul 31;16(15):2180. doi: 10.3390/polym16152180 (PMC11314156; doi:10.3390/polym16152180)
Supplement: Supplementary file 1 [file polymers-16-02180-s001.zip › polymers-3120223-supplementary.pdf]

polymers

Supplementary

Table S1. The results of the tribological tests of the PES-based composites. The 'block-on-ring' scheme. The GCr15 counterpart; P = 60 N; V = 0.3 m/s.

No.	Composition, wt.%*	CoF	WR, $mm^3/N{\cdot}m$, 10^{-6}	Temperature, *T*, °C
1	PES/10SCF (*l*=53 µm)	0.447±0.062	66.74±5.69	29.87±1.74
2	PES/10SCF (*l*=100 µm)	0.411±0.050	63.15±1.24	27.52±0.63
3	PES/10SCF (*l*=200 µm)/ 0.5CNF (∅60 nm, *l*=2 mm)	0.450±0.053	51.74±1.619	23.19±1.45
4	PES/10SCF (*l*=200 µm)/ 10Gr (∅4 µm)	0.328±0.039	11.39±1.29	23.75±0.88
5	PES/10SCF (*l*=200 µm)/ 10PTFE (5 µm)	0.340±0.022	25.79±2.98	27.13±0.84
6	PES/10SCF (*l*=200 µm)/ 1 nano SiO_2(∅50 nm)	0.449±0.055	63.21±1.60	27.31±1.00
7	PES/10SCF (*l*=2 mm)/ 10Gr (∅4 µm)	0.323±0.028	10.44±0.41	27.87±1.20
8	PES/5CF (*l*=200 µm)/ 5CF (*l*=2 mm)	0.412±0.034	54.57±1.01	28.39±1.44
9	PES/10SCF (*l*=2 mm)/ 10PTFE (∅5 µm)	0.320±0.021	14.43±1.28	26.23±0.99
10	PES/10SCF (*l*=2 mm)/ 1 nano SiO_2(∅50 nm)	0.438±0.047	50.08±1.41	26.89±1.26
11	PES/20SCF (*l*=2 mm)/ 1MoS_2 (∅4 µm)	0.523±0.034	66.16±2.94	24.99±0.78
12	PES/20SCF (*l*=2 mm)/ 1 HNT (∅50 nm, *l*=2 mm)	0.495±0.021	60.21±1.38	24.49±1.01
13	PES/20SCF (*l*=2 mm)/ 10 Gr (∅4 µm)	0.350±0.036	18.83±0.46	25.49±0.90
14	PES/20SCF (*l*=2 mm) / 1 nano SiO_2 (∅50 nm)	0.457±0.045	35.88±2.99	26.67±1.45
15	PES/20SCF (*l*=2 mm) / 1 SiC (∅80 nm)	0.282±0.096	47.44±7.01	26.55±0.63

* *l* is length, ∅ is diameter, SCF is short carbon fibers, CNF is carbon nano fibers, PTFE is polytetrafluoroethylene, Gr is graphite, CF is carbon fibers, HNT is halloysite nano tubes
